# Development and Standardization of a High-Throughput Multiplex Immunoassay for the Simultaneous Quantification of Specific Antibodies to Five Respiratory Syncytial Virus Proteins

**DOI:** 10.1128/mSphere.00236-19

**Published:** 2019-04-24

**Authors:** Rutger M. Schepp, Cornelis A. M. de Haan, Deidre Wilkins, Hans Layman, Barney S. Graham, Mark T. Esser, Guy A. M. Berbers

**Affiliations:** a Center of Infectious Disease Control, National Institute of Public Health and the Environment (RIVM), Bilthoven, the Netherlands; b Virology Division, Department of Infectious Diseases and Immunology, Faculty of Veterinary Medicine, Utrecht University, Utrecht, the Netherlands; c AstraZeneca, Gaithersburg, Maryland, USA; d Vaccine Research Center, National Institute of Allergy and Infectious Diseases, National Institutes of Health, Bethesda, Maryland, USA; University of Maryland School of Medicine

**Keywords:** assay development, immunoassays, multiplex, respiratory syncytial virus

## Abstract

Human respiratory syncytial virus (RSV) is a major cause of severe respiratory disease in (premature) newborns and causes respiratory illness in the elderly. Different monoclonal antibody (MAb) and vaccine candidates are in development worldwide and will hopefully become available within the near future. To implement such RSV vaccines, adequate decisions about immunization schedules and the different target group(s) need to be made, for which the assessment of antibody levels against RSV is essential. To survey RSV antigen-specific antibody levels, we developed a serological multiplex immunoassay (MIA) that determines and distinguishes antibodies against the five RSV glycoproteins postfusion F, prefusion F, Ga, Gb, and N simultaneously. The standardized RSV pentaplex MIA is sensitive, highly reproducible, and specific for the five RSV proteins. The preservation of the conformational structure of the immunodominant site Ø of prefusion F after conjugation to the beads has been confirmed. Importantly, good correlation is obtained between the microneutralization test and the MIA for all five proteins, resulting in an arbitrarily chosen cutoff value of prefusion F antibody levels for seropositivity in the microneutralization assay. The wide dynamic range requiring only two serum sample dilutions makes the RSV-MIA a high-throughput assay very suitable for (large-scale) serosurveillance and vaccine clinical studies.

**IMPORTANCE** In view of vaccine and monoclonal development to reduce hospitalization and death due to lower respiratory tract infection caused by RSV, assessment of antibody levels against RSV is essential. This newly developed multiplex immunoassay is able to measure antibody levels against five RSV proteins simultaneously. This can provide valuable insight into the dynamics of (maternal) antibody levels and RSV infection in infants and toddlers during the first few years of life, when primary RSV infection occurs.

## Abstract (precis)

In view of vaccine and monoclonal development to reduce hospitalization and death due to lower respiratory tract infection caused by RSV, assessment of antibody levels against RSV is essential. This newly developed multiplex immunoassay is able to measure antibody levels against five RSV proteins simultaneously. This can provide valuable insight into the dynamics of (maternal) antibody levels and RSV infection in infants and toddlers during the first few years of life, when primary RSV infection occurs.

## INTRODUCTION

*Respiratory syncytial virus* (RSV) is an enveloped, negative-strand RNA virus and a member of the *Pneumoviridae* family. RSV can cause acute lower respiratory tract infection (ALRI), mostly in infants <5 years of age but also in the elderly and in immunocompromised individuals (1). RSV infection is the most common cause of hospital admission and death from ALRI in (preterm) infants and is associated with high health care costs (2, 3). Although protection from RSV infection is not completely understood, neutralizing antibodies present at or above a protective threshold (4) are assumed to prevent infection and serious disease. Passive immunization with high-titer intravenous immunoglobulin (IVIG) or RSV-neutralizing monoclonal antibodies (MAbs) reduces serious disease caused by RSV (5). Different RSV MAbs and vaccine candidates are in development worldwide and will hopefully become available within the next decade (6, 7). The future RSV vaccines have to protect newborns, infants, or the elderly from disease, and for vaccine efficacy trials the assessment of specific antibody levels against RSV proteins is essential for vaccine evaluation and the diagnosis of infection. In addition, for adequate decisions about RSV vaccine immunization schedules, as well as the different target groups for immunization, a rapid and high-throughput assay compared to the more labor-intensive neutralization test (NT) would be beneficial. Multiplex assays have these advantages compared to (commercially available) enzyme-linked immunosorbent assay (ELISA) and the NT, making them highly suitable for testing large numbers of samples from vaccine clinical trials and serosurveillance studies. Therefore, we have developed and standardized a fluorescent, bead-based, multiplex immunoassay (MIA) for simultaneous quantitative analysis of antibodies directed against the five RSV-specific glycoproteins postfusion F, prefusion F, Ga and Gb, and nucleoprotein N. The aim of this study was to develop an RSV-specific multiplex assay that not only quantitatively detects antibodies directed against the major (glyco)proteins of the virus but also provides data complementary to the data provided by the NT. Therefore, we compared the RSV-MIA with a microneutralization assay (MN).

## RESULTS

### Development of the RSV-MIA.

For the five RSV antigens, the conjugation concentration was determined starting with 5 µg/1.2 × 10^6^ beads (100 µl). Raising the concentration to 20 µg/1.2 × 10^6^ beads did not result in significantly higher mean fluorescence intensities (MFIs) for postfusion F, prefusion F, and nucleoprotein, but for Ga and Gb the MFIs increased by approximately 50%. Increasing the concentration of Ga and Gb even further, up to 40 µg, did not increase the MFIs any further. Therefore, we selected 5-µg/100-µl bead suspensions for prefusion F, postfusion F, and nucleoprotein and 20-µg/100-µl bead suspensions for Ga and Gb as the conjugation concentration. The standard assay buffer used for other protein and virus MIAs developed in our lab (8–10) consisting of phosphate-buffered saline (PBS; pH 7.2), 0.1% (vol/vol) Tween 20, and 1% (wt/vol) bovine serum albumin (BSA) proved again to be optimal for the RSV pentaplex immunoassay (RSV-MIA).

### Performance of the reference sera.

**FIG 1:**
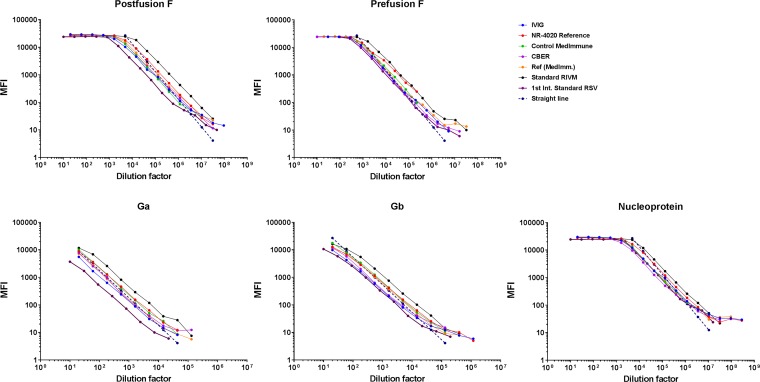
Linearity and range of standard sera. Seven sera were serially diluted to assess parallelism (dilutional linearity) and the dynamic range of the RSV-MIA for postfusion F, prefusion F, Ga, Gb, and N proteins.

**FIG 2:**
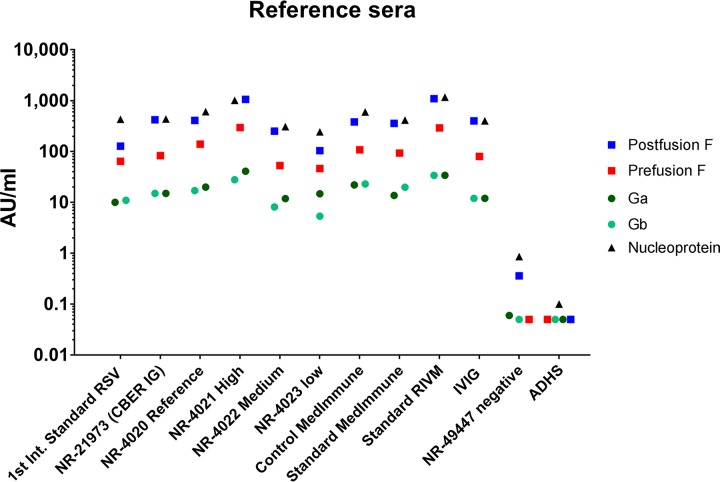
Potency of reference and control sera. The potency of different standard and control sera was established using intraveneous immunoglobulin (IVIG) as a reference with assigned potencies of 400, 80, 12, 12, and 400 AU/ml for the postfusion F, prefusion F, Ga, Gb, and N proteins, respectively.

The performance of the six reference sera and one control sample was tested in the RSV-MIA, including the reference standards from MedImmune, CBER, the National Institute for Biological Standards and Control (NIBSC; first international standard, RSV16/344), American Type Culture Collection (ATCC; NR-4020), RIVM (in-house), and IVIG. Diluting these standards up to 10^8^ for prefusion F, postfusion F, and nucleoprotein and up to 10^6^ for Ga and Gb resulted in a dynamic range of 4 log_10_ (10,000) for all RSV antigens (Fig. 1). Within this dynamic range, the lines show very good parallelism to the ideal line, with only small deviations at very high dilutions. Based on the magnitude of the MFI signals of these reference standard sera, we arbitrarily assigned to the IVIG sample the potencies of 400, 80, 12, 12, and 400 arbitrary units (AU)/ml for the postfusion F, prefusion F, Ga, Gb, and N proteins, respectively. Using IVIG as a reference, the other six reference standards and four control sera (from the NR panel and Medimmune) were converted to AU/ml for all five RSV antigens, resulting in the highest antibody levels to postfusion F and N, followed by prefusion F 3 to 10 times lower and approximately again 10 times lower for Ga and Gb (Fig. 2). The negative controls had concentrations near or even below the lower limit of quantification (LLOQ) at the 1/200 dilution.

### Specificity of the RSV MIA.

**FIG 3:**
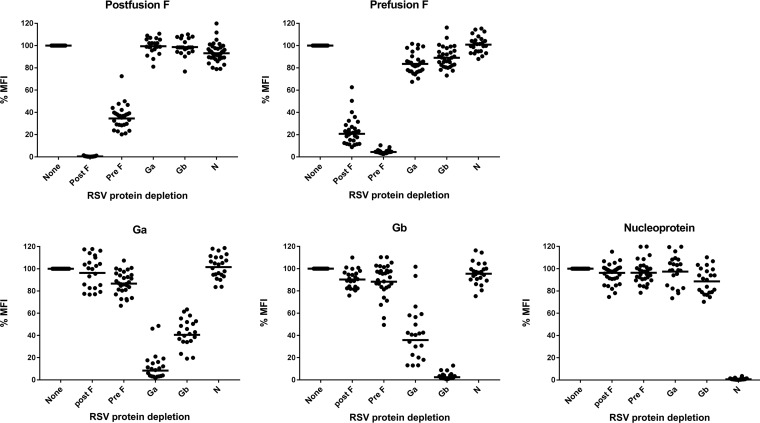
Specificity illustrated as inhibition with homologous and heterologous RSV proteins. Specificity of the five RSV proteins was assessed by depleting sera with an excess of homologous or heterologous proteins of the pentaplex MIA and compared to an uninhibited benchmark (100% MFI).

**FIG 4:**
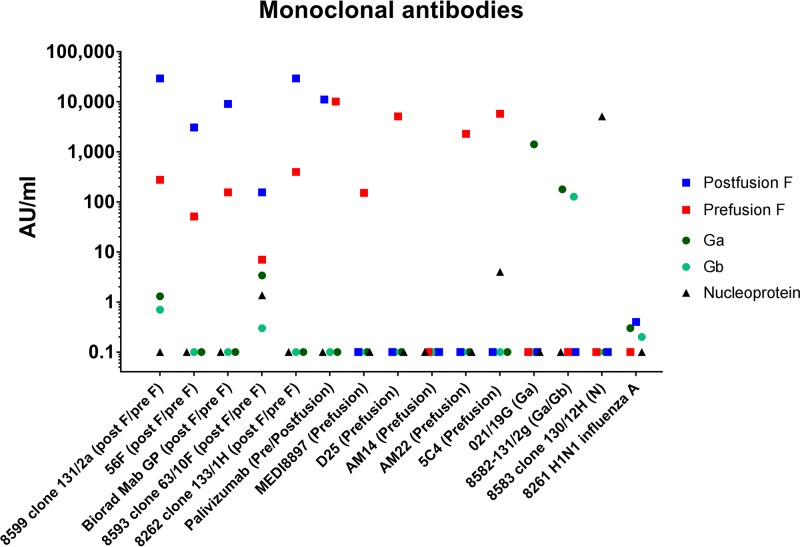
Specificity of MAbs. Specific MAbs were used to assess the conformation and cross-reactivity of five different RSV-MIA proteins after conjugation to beads.

Between 25 and 40 serum samples with variable levels of IgG to each of the five RSV proteins were tested for antigen specificity in inhibition experiments, and the outcome is illustrated in Fig. 3 The homologous inhibition varied between 92% (Ga) and 99% (postfusion F) as summarized in Table 1 . The heterologous inhibition amounted to ≤16% for all five RSV antigens, except between postfusion F and prefusion F (79 and 65%) and between Ga and Gb (64 and 59%) due to the considerable structural homology between these RSV glycoproteins. To confirm that the five different RSV antigens could be measured in multiplex form despite this homology between the F and G proteins, we evaluated a subset of the sera from the validation panel in assays using monoplex bead sets and pentaplex bead sets. The comparison between the monoplex and the multiplex MIA showed very good correlations (*r*^2^ varying between 0.95 and 0.99; see Fig. S1 in the supplemental material), showing no influence on the MFI signals due to the homology. Furthermore, the specificity (purity) and the conformation of the RSV proteins were further investigated with a set of RSV-specific MAbs (Table S1). As illustrated in Fig. 4, the MAbs detected only their specific RSV protein(s), as indicated in the literature, except for maybe MAb 8599 clone131/2a, which bound both postfusion F and prefusion F, and MAb 8593 clone 63/10F, which bound to both F proteins and also Ga, Gb, and N slightly. Palivizimab recognized both postfusion F and prefusion F as expected (11), as well as Bio-Rad MAb GP, 56F, and 8262 clone 133/1H. The MAbs MedI 8897, D25, AM22, and 5C4 all bound only the prefusion F antigen, thereby confirming that the site Ø (zero) region was retained on the prefusion F conjugated to the beads (12). MAb 5C4 showed <1,000-fold lower binding to N. Interestingly, MAb AM14 did not bind any of the RSV proteins, including the DS-Cav1 form of prefusion F. The three MAbs against Ga, Gb, and N all showed high specificity to their respective antigens.

**TABLE 1:** Specificity of the pentaplex RSV-MIA by percent inhibition of the homologous and heterologous RSV proteins in the MIA

RSV protein	% inhibition				
	Postfusion F	Prefusion F	Ga	Gb	N
Postfusion F	99	65	1	1	7
Prefusion F	79	96	16	11	0
Ga	4	13	92	59	0
Gb	10	12	64	98	5
N	4	3	2	11	99

10.1128/mSphere.00236-19.1 FIG S1 Monoplex versus multiplex assay illustrated as scatterplots: interference between the five RSV-MIA proteins was evaluated in a monoplex versus multiplex setup. Download FIG S1, TIF file, 0.7 MB. Copyright © 2019 Schepp et al. 2019 Schepp et al. This content is distributed under the terms of the Creative Commons Attribution 4.0 International license.

10.1128/mSphere.00236-19.3 TABLE S1 Characteristics of the monoclonal antibodies. Download Table S1, DOCX file, 0.02 MB. Copyright © 2019 Schepp et al. 2019 Schepp et al. This content is distributed under the terms of the Creative Commons Attribution 4.0 International license.

### Validation of the RSV MIA.

**FIG 5:**
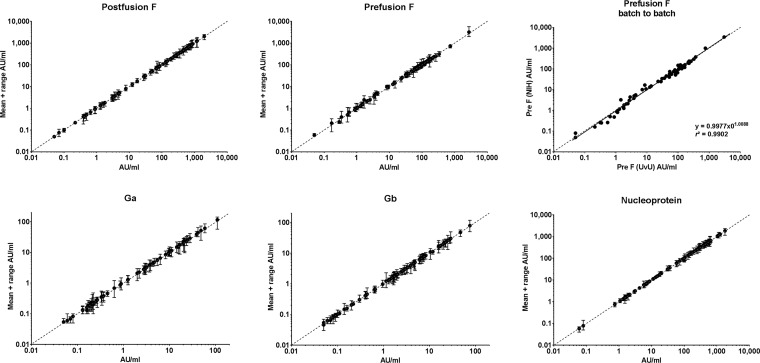
Interassay variation illustrated in scatterplots, including the batch-to-batch comparison for prefusion F. Samples were tested independendly five times over 2 months using two different bead batches. The scatterplots (log-log) depict the variation (mean + range) of the assay for all RSV-MIA proteins.

The precision in terms of repeatability of the RSV-MIA was assessed by intra-assay variation (Fig. S2), and the intermediate precision was assessed by interassay variation over five assay runs within 4 weeks using two separate antigen-bead lots (Fig. 5). The mean percent coefficient of variation (%CV) for the intra-assay variation ranged from 3 to 13%, and for the interassay variation it ranged from 16 and 26% (Table 2). The higher percentages found for Ga and Gb were due to the relatively high number of sera with a low concentration of antibodies to these proteins present in the serum panels. The correlations between the different assay runs revealed an *r*^2^ of >0.98 in all cases (data not shown). In Fig. 5, the batch-to-batch variation between the two different prefusion F preparations was very good (*r*^2^ = 0.99).

**TABLE 2:** Intra- and interassay variation; precision of the RSV-MIA represented as the %CV over five independent separate runs (interassay) and the precision of two dependent runs within 1 day (intra-assay)

Variation	%CV				
	Postfusion F	Prefusion F	Ga	Gb	N
Intra-assay	4	4	13	9	3
Interassay	20	16	26	23	19

10.1128/mSphere.00236-19.2 FIG S2 Intra-assay variation of the RSV-MIA illustrated as scatter plots: samples were tested with the same sample dilutions on the same day on separate plates. Download FIG S2, TIF file, 0.7 MB. Copyright © 2019 Schepp et al. 2019 Schepp et al. This content is distributed under the terms of the Creative Commons Attribution 4.0 International license.

The dynamic range of the different reference curves was used to determine the LLOQ using conservative lower IgG concentration limits. The low level of nonspecific binding of the secondary detection antibody observed at the lower part of the curves resulted in low LLOQs that varied from 0.12 mAU/ml for prefusion F to 0.69 mAU/ml for Ga (Table 3). In practice, adjusting for a 200-fold serum dilution results in LLOQs between 0.021 and 0.138 AU/ml (Table 3). Different assay runs using the serum panel and all prefusion F MAbs over time with the same bead sets showed that RSV-conjugated microspheres stored at 4°C remained stable for at least 6 months up to 1 year without a significant reduction in MFI signal and a reproducibility of *r*^2^ = 0.97 to 0.99 (data not shown).

**TABLE 3:** Calculated LLOQ for each of the five RSV proteins in the MIA and also expressed in a 200-fold serum dilution

Parameter	Mean ± SD				
	Postfusion F	Prefusion F	Ga	Gb	N
LLOQ (mAU/ml)	0.106 ± 0.161	0.123 ± 0.059	0.688 ± 0.349	0.684 ± 0.380	0.265 ± 0.417
200-fold dilution (AU/ml)	0.021 ± 0.032	0.025 ± 0.012	0.138 ± 0.070	0.137 ± 0.068	0.053 ± 0.083

### Comparison with an RSV neutralization assay.

**FIG 6:**
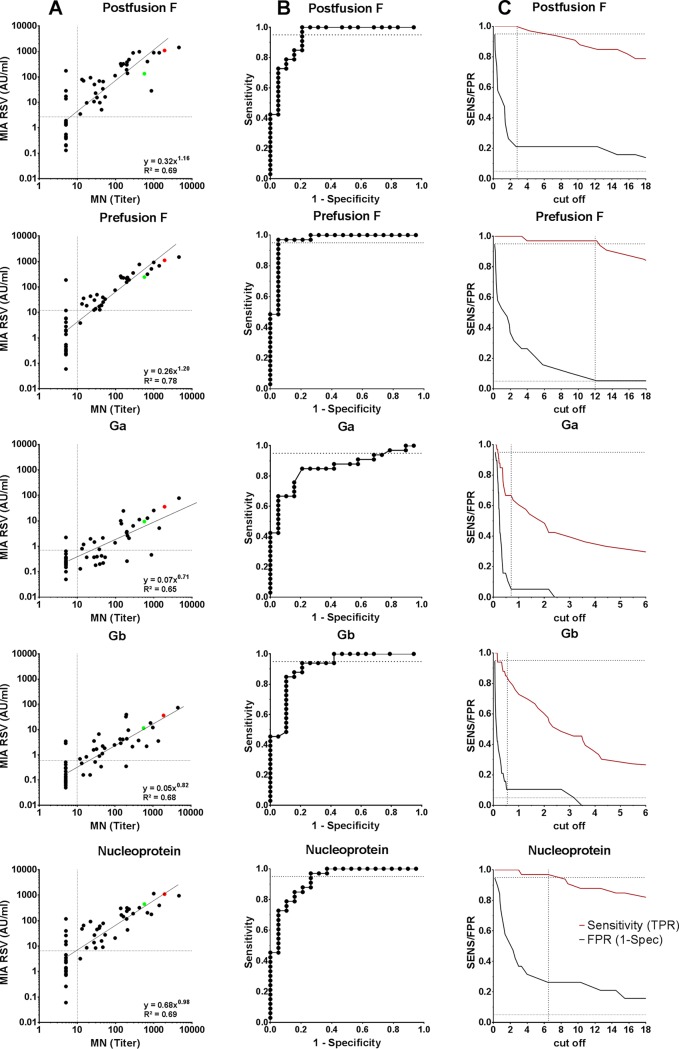
Correlation between MIA and microneutralization (MN). (A) Scatterplots of the comparison between the MN assay (titers) and the RSV-MIA (AU/ml) for five RSV proteins (depicted separately). The vertical dotted line indicates the LLOQ of 1:10 in the MN. Samples below the LLOQ were assigned a titer of 1:5. The horizontal lines represent the estimated optimal cutoffs as a surrogate of positivity in the MN for the five RSV proteins evaluated in panel C. (B) ROC curves for the five RSV proteins plotting the sensitivity against the false-positive rate (1 – specificity) at various cutoff settings indicating the AUC. (C) ROC curves plotting the false-positive rate (FPR or 1 – specificity) and true-positive rate (TPR or sensitivity) as a function of threshold values. The dotted horizontal lines represent 95% sensitivity, and the vertical lines indicate the chosen cutoff considered an optimal trade-off between the true and false positivity rates.

The additional serum panel composed of 52 samples with a wide concentration range in the MIA was tested in a microneutralization assay (MN), and the results from both assays compared for all five antigens. Linear regressions revealed good comparisons for the five RSV proteins (Fig. 6A), with Pearson *r* values varying between 0.81 and 0.88 (Table 4). The best correlation was obtained for prefusion F, with only one sample positive in the MIA and negative in the MN. We found that there were three and four samples that were positive in the MIA and <LLOQ in the MN for postfusion F and N, respectively. For Ga and Gb, the graphs are more scattered, resulting in more outliers, which also include samples with negative MIA and positive MN levels. The Pearson *r* for prefusion F was 0.93 when the outlier was discarded. The comparison between the MIA and the MN was also analyzed by receiver operating characteristics (ROC) curves to estimate an MIA value that correlated with positivity in the RSV MN (Fig. 6B and C). The estimated ROC curves indicated low false-positive rates (FPRs) and high sensitivities over a wide range for all five RSV proteins, with the lowest area under curve (AUC) for Ga and the highest AUC for prefusion F (Fig. 6B and Table 4). On basis of the comparison between sensitivity and specificity (1 – FPR), a cutoff value that identified RSV MN-positive samples in antigen-specific AU/ml could be estimated for the five RSV proteins (Fig. 6C and Table 4).

**TABLE 4:** Characterisitics of the correlation and ROC curves from comparison between MIA and MN

Parameter	Postfusion F	Prefusion F	Ga	Gb	N
Optimal cutoff MIA (AU/ml)	2.70	12.00	0.70	0.60	6.50
AUC	0.94	0.97	0.85	0.92	0.93
True-positive rate (sensitivity)	1.00	0.97	0.67	0.82	0.97
False-positive rate (1 – specificity)	0.21	0.05	0.05	0.11	0.26
Pearson *r*	0.83	0.88	0.81	0.82	0.83
Slope	1.16	1.20	0.71	0.82	0.98
*y* intercept	–0.49	–0.59	–1.15	–1.28	–0.17

## DISCUSSION

Here we describe the development and standardization of a fluorescent, bead-based multiplex immunoassay for the simultaneous detection of IgG antibodies against five specific RSV proteins for use in vaccine and MAb clinical trials and large serosurveillance studies. The pentavalent RSV-MIA showed a high specificity for the five conjugated RSV proteins, i.e., the postfusion F, prefusion F, Ga, Gb, and N proteins. The MIA proved to be highly sensitive and reproducible, with a bead stability of up to 1 year. Importantly, this RSV-MIA showed a good correlation with the microneutralization assay for all five RSV proteins, but especially for prefusion F.

All the reference or standard sera used in this study (six in total) and also the high and medium control sera (three in total) showed IgG antibody levels similar to each of the five RSV antigens, providing a large dynamic range of the assay (4 log_10_ dilutions) and indicating that these standards are more or less interchangeable. The highest MFI signals (in the plateau of the curve) were found for postfusion F and remarkably also for N. Although N is an RSV nucleocapsid protein, the prevalence of N-specific antibodies in the circulation is apparently quite similar in all of these sera to the other four RSV proteins. The MFI signals against Ga and Gb were 10-fold lower than those for the postfusion F, prefusion F, and N proteins, indicating that these RSV proteins are probably less immunogenic, resulting in lower antibody levels in the circulation. Another explanation could be that the large amount of carbohydrates and/or the low lysine content in the G proteins negatively influences the conjugation of these proteins to the beads.

The first international standard (NIBSC RSV 16/344) was assigned 1,000 IU/ml for use in an RSV neutralization assay against the whole virion (13). Because no reference standard is available for the separate RSV proteins, we assigned arbitrary units to the easily available IVIG preparation at the beginning of the RSV-MIA development, reflecting the observed MFI signals. During the RSV-MIA development process, we composed our in-house standard, which we calibrated during the study to the IVIG preparation and the international standard. In the future, we will be using our in-house reference because it is composed of several human sera with high RSV-specific antibody levels and not of purified and lyophilized IgG, as in the case of IVIG, thereby being more reflective of serum samples that we will test.

Despite the amino acid homology between postfusion F and prefusion F within the virus and between Ga and Gb proteins of different virus strains, no influence of cross-reactivity was observed when sera were measured in the multiplex versus monoplex assays. The sequence homology of the G protein genotypes within the same subgroup (RSV A or B) is around 80%, while the homology of the G protein between RSV A and B serogroups is approximately 50% (14). Between postfusion F and prefusion F, only conformational differences exist; however, to stabilize the prefusion F, conformation 4 amino acid mutations have been applied to establish DS-Cav1, which resulted in a 0.7% sequence difference (15). Depletion with Ga or Gb led to a reduction of approximately 60% of the Gb- or Ga-specific antibodies, respectively, indicating that a substantial amount of Ga- and Gb-specific antibodies bind shared epitopes on Ga and Gb, which would likely be in the central conserved domain. For postfusion F approximately 65% of the antibodies could be adsorbed by prefusion F, and for prefusion F up to 80% could be adsorbed with postfusion F, showing the existence of shared epitopes on postfusion F and prefusion F. It should be mentioned though that considerable variation was found between the different human samples, suggesting that each individual has strain-specific and epitope-specific immune responses (16).

The nine MAbs recognizing post- and prefusion F, Ga, Ga/Gb, or N all showed binding as expected, with the exception of MAb 8599 clone131/2a and MAb 8593 clone 63/10F. MAb 8599 clone 131/2a bound to postfusion F but also to prefusion F, although 100× lower and very slightly with Ga and Gb (1 AU/ml). While the weak binding to Ga and Gb can be attributed to nonspecific binding, the reaction with prefusion F is contradictory to the literature, although binding to site I to a limited extent was observed earlier (17–19). MAb 8593 clone 63/10F showed not only low responses to Ga, Gb, and N but also similarly a low response against prefusion F and only a 20-fold-higher response against postfusion F, proving its lack of specificity. The three MAbs D25, AM22, and 5C4 all bind to the antigenic site Ø (zero) on the membrane-distal apex of the prefusion F trimer, and all have high neutralizing activity (20), whereas AM14 is specific for cleaved trimeric prefusion F and to a limited extent for the uncleaved monomeric prefusion F recognizing a quaternary epitope (site V) (21, 22). All MAbs to the unique epitopes on site Ø of prefusion F bound as expected (20, 21). AM14 did not show specific binding to site V of prefusion F, suggesting there may be some separation of protomers within the prefusion F trimer on the beads while maintaining the untriggered prefusion conformation based on the site Ø binding within each monomer. This means that some antibodies binding quaternary epitopes might be missed in this assay, but the majority of the conformation-dependent responses are being measured, and that the form of preF on the bead is in a metastable conformation with site Ø retained after conjugation to the beads. This conformation of DS-Cav1 remained stable for up to 1 year upon storage at 4°C.

Importantly, the correlation between the RSV MN and the MIA for each of the five RSV antigens, including the Ga, Gb, and N proteins, was very good. The correlation of the neutralization titer with the antibody levels to prefusion F yielded with high sensitivity (97%) and specificity (95%) an arbitrary cutoff value of 12 AU/ml for RSV MN positivity. The only outlier in the prefusion F/MN comparison was a sample from a 3-year-old child that was negative in the MN but clearly positive in the MIA for all five RSV proteins. We cannot explain this mismatch, because a healthy child of 3 years is expected to have been infected with RSV, resulting in neutralizing antibodies in the circulation. One possibility is that the levels of RSV-specific antibodies in this child as measured in the MIA have not reached the threshold for virus neutralization. Nevertheless, this comparison shows that the RSV multiplex assay can complement the neutralization assay in large serosurveillance and vaccine studies.

An important advantage of the MIA over MN and ELISA is the 4-log linear range, allowing the reliable quantitation of samples over a wider concentration range and reducing the need of (re)testing samples at a series of different dilutions. The chosen dilution series of the reference enabled just two dilutions of the serum samples (1/200 and 1/8,000) to sufficiently cover a broad range of antibody concentrations, thereby providing a high-throughput assay. It has been shown that performance of the MIA is more cost-effective than ELISA (and certainly more than a neutralization assay) when at least three different antigens are measured in one assay (8). In addition, the pentaplex RSV-MIA requires less serum (5 µl) and a smaller amount of antigen compared to the ELISA and neutralization assays, including MN. Another advantage of the RSV MIA is that it can be easily modified to measure RSV Ag-specific IgM and IgA and for testing other matrices like nasal fluid and breast milk. These modified assays may be useful in better understanding the immune response to RSV and identifying a correlate of protection.

A limitation of the pentaplex RSV-MIA is the availability of the antigens. Although postfusion F, Ga, and Gb are commercially available, prefusion F and nucleoprotein are not and can be obtained by donation from some institutes or produced as recombinant proteins in expression systems. In addition, RSV A and B viruses continue to evolve, and newer versions of Ga and Gb may be required to have antigens that are reflective of RSV strains currently in circulation.

In conclusion, we developed a specific, sensitive, reproducible, sample- and antigen-saving method for simultaneous quantitative measurement of IgG antibodies directed against five different RSV proteins. Furthermore, the MIA showed a good correlation with an RSV neutralization assay for all five RSV proteins, resulting in an arbitrary cutoff value of prefusion F antibody levels for seropositivity in the neutralization assay. The pentaplex RSV-MIA is a good alternative to ELISA and of complementary value for the neutralization assay in (large-scale) immunosurveillance studies and vaccine (MAb) clinical trials.

## MATERIALS AND METHODS

### Serum samples.

For the development and standardization of the RSV-MIA, a panel of 80 serum samples from multiple sources was used. The panel consisted of cord blood sera obtained from Radboud University Nijmegen (*n* = 20) (23), a panel of human antiserum and immunoglobulin to RSV from CBER (NR-32832; BEI Resources, National Institute of Allergy and Infectious Disease [NIAID], National Institutes of Health [NIH], Manassas, VA) (*n* = 6), sera from infants 8, 11, and 12 months old who participated in a pneumococcal vaccination study (*n* = 32) (24), and sera from 9-year-old children who participated in a pertussis vaccination study (*n* = 16) (25). An RSV reference serum (MedImmune RSV12) and control serum were obtained from MedImmune (Gaithersburg, MD). Intravenous immunoglobulin (IVIG; Baxter LE-07-32443; 10 mg/ml), used as reference in this study, as well as antibody-depleted human sera (ADHS; HS1200 [Valley Biomedical, Winchester, VA]) (*n* = 2) and fetal calf serum (HyClone [Fischer Scientific]), completed the panel. In addition, the first international RSV standard was obtained from the NIBSC (RSV 16/344; NIBSC, Potters Bar, UK), and an in-house reference serum was composed of sera (*n* = 6) with a high antibody concentration against all RSV proteins for application as an in-house standard in the future. For comparison of the MIA with the microneutralization test (MN), an additional serum panel (*n* = 52) was composed of samples from children who participated in the same studies described above (5 to 12 months old [*n* = 32], 3 years old [*n* = 8], and 9 years old [*n* = 8]), supplemented with the first international RSV standard (NIBSC), the in-house standard (RIVM), a high positive cord blood sample, and ADHS as a negative control.

### Antigens and MAbs.

The RSV glycoproteins postfusion F, Ga, and Gb and the nucleoprotein N were kindly donated by AstraZeneca/MedImmune (26), and a small amount of prefusion F (DS-Cav1 form) was kindly donated by Xander de Haan (Utrecht University, Utrecht, the Netherlands) (17). During the development of the RSV-MIA, a larger amount of prefusion F (DS-Cav1 form, subtype A) (15) was obtained from Barney Graham (NIH, Bethesda, MD). The MAbs 8262 clone 133-1H, 8593 clone 63-10F, 8599 clone 131-2A, 8582 clone 131-2G, and 858-3 clone 130-12H were obtained from Chemicon (EMD Millipore). MAb GP clone 8C5 was obtained from Bio-Rad, and MAbs 56F and 021/19 G were obtained as hybridoma supernatant from Oliver Wicht (27), all of murine origin. The MAbs D25, palivizumab, and MEDI 8897 were donated by MedImmune, and the MAbs 5C4, AM14, and AM22 were obtained from Barney Graham; all were of human origin except the 5C4 murine MAb. The characteristics and specificity of these MAbs are summarized in Table S1.

### Procedure for the RSV pentaplex immunoassay.

Each of the five RSV antigens were coupled to distinct color-coded activated carboxylated beads (Bio-Rad Laboratories, Hercules, CA) by using a two-step carbodiimide reaction as described previously (8). After the beads (12.5 × 10^6^ beads/ml) were washed in PBS (pH 7.2), they were activated by the addition of 5 mg of 1-ethyl-3-(-3-dimethylaminopropyl)-carbodiimide hydrochloride (EDC; Thermo Fisher Scientific) and 5 mg of *N*-hydroxy-sulfosuccinimide (sulfo-NHS; Thermo Fisher Scientific) in 1 ml of PBS. After activation, the beads were washed in PBS and resuspended in 1 ml of PBS containing 50 µg of postfusion F protein, prefusion F protein, or nucleoprotein N or 200 µg of Ga protein and Gb protein. The beads were incubated for 2 h at room temperature in the dark under constant rotation, washed three times with PBS, and stored in the dark in PBS containing 0.03% (wt/vol) sodium azide, 0.05% Tween 20, and 1% (wt/vol) bovine serum albumin at 4°C until use.

The assessment of the RSV antigen-specific antibody concentration in the serum samples was performed essentially as described previously (8). The reference standard serum, serum samples, and control sera were diluted in PBS (pH 7.2) containing 0.1% (vol/vol) Tween 20 and 1% (wt/vol) BSA. Blanks were also included on each plate. The reference standard (IVIG) was serially diluted 3-fold over 12 wells (1/20 starting dilution). Serum samples were measured in two dilutions (1/200 and 1/8,000). After washing, the beads were incubated with R-phycoerythrin-labeled goat anti-human IgG antibody that was Fcγ fragment specific (Jackson Immunoresearch Laboratories, West-Grove, PA). After the final washing steps, the beads were resuspended in 100 μl of PBS and shaken at 600 rpm on a platform shaker. The measurement of the samples was performed using a Bio-Plex 200 in combination with Bio-Plex Manager software version 6.1 (Bio-Rad Laboratories). For each analyte, the MFI was converted to AU/ml by interpolation from a five-parameter logistic standard curve. During the development and standardization of the RSV-MIA, the IVIG was used as a reference standard throughout all experiments. The prefusion F preparation from Xander de Haan was also used throughout development, except for the inhibition experiments, where prefusion F from Barney Graham was used.

### Specificity of the RSV-MIA.

The specificity of the MIA was assessed by competitive-inhibition experiments. Homologous and heterologous inhibition was performed with the five RSV-specific proteins using a subset of the validation panel (*n* = 40). Depending on antibody levels to each of the five antigens, the number of samples in these inhibition experiments varied between 25 and 40. The serum samples were prediluted 1/2,000 for the postfusion F, prefusion F, and N competition experiments or 1/100 for Ga and Gb in assay buffer (PBS containing 0.1% [vol/vol] Tween 20 and 1% [wt/vol] BSA). Samples were subsequently mixed 1:1 (vol/vol) with each of the five RSV proteins (50 µg/ml) separately in assay buffer and incubated for 1 h at room temperature. Serum dilutions of 1/4,000 (postfusion F, prefusion F, and N) and 1/200 (Ga and Gb) without RSV antigens were used as a benchmark (100%). Homologous and heterologous inhibition was expressed as the percent reduction in each RSV protein-specific IgG antibody concentration relative to the benchmark.

The set of MAbs was used to confirm the specificity of the antigens on the beads and to determine any possible changes in conformation of the antigens after conjugation to the beads, especially for prefusion F. Furthermore, the possible interference between the five different bead sets was investigated by comparing IgG concentrations generated from the monoplex MIA with the pentaplex MIA using a subset of the validation panel (*n* = 40).

### Validation of the RSV-MIA.

For the RSV-MIA validation, the dilutional linearity, the precision, and the range of the assay were assessed. In addition, the bead stability and the correlation of the RSV-MIA with the neutralization assay were determined.

The dilutional linearity of the RSV multiplex assay was established with seven different samples, including the references from MedImmune, CBER, RIVM (in-house), NIBSC (1st international standard) and ATCC (NR-4020), IVIG, and a control serum (Medimmune) with known high antibody concentrations against the RSV proteins. These sera were tested in a 3-fold serial dilution series to determine the dynamic range of the assay. The results of the measurements were incorporated in log-log graphs using the five-parameter logistic fit curve.

The LLOQ, defined as the lowest concentration of RSV-specific antibodies that can be quantified reliably (%CV < 20%), was calculated as the average lowest concentration of the reference sera detected at the lower limit of linearity of the reference standard curves in 10 measurements.

The precision of the assay was established for all five RSV proteins—postfusion F, prefusion F, Ga, Gb, and N—using the subset panel (*n* = 40) for the intra-assay variation (repeatability) and the whole validation panel minus two negative controls (*n* = 78) for the interassay variation (intermediate precision). For the intra-assay variation, the 40 individual samples were analyzed on the same day on two independent plates. For the interassay variation, the 78 samples were tested in five independent separate assay runs over a period of 4 weeks using two different antigen-bead lots. For the precision assays, the coefficient of variation (%CV) between each of these results for all five antigens was calculated, and the correlation coefficients (*R*^2^) were determined.

For comparison with an RSV microneutralization assay (MN), the additional sample set (*n* = 52) was measured in a MedImmune RSV microneutralization test performed at BioAgilytix (Durham, NC) as described earlier using a recombinant RSV-A2-GFP virus strain (28), and the log_2_ 50% inhibitory concentration MN titer (fold dilution) for each sample was calculated. The correlation between the MIA and MN was analyzed with Pearson’s correlation on log-transformed concentrations (MIA) and titers (MN) with the correlation coefficient *r*^2^, including the data of the lower limit of the MN (<3.32 log_2_ = 1 log_10_). The samples with titers below the lower limit of the MN were arbitrarily assigned to 0.5 log_10_. Assuming that MN titers above the lower limit are true-positive ROC curves, plotting the assay sensitivity (true positive rate) in the function of the true negative rate (1 – specificity) for different cutoff points was used to determine the optimal threshold for RSV proteins in the MIA serology assay.
