# Supplementary figures and images for: Development and Standardization of a High-Throughput Multiplex Immunoassay for the Simultaneous Quantification of Specific Antibodies to Five Respiratory Syncytial Virus Proteins
Source: mSphere. 2019 Apr 24;4(2):e00236-19. doi: 10.1128/mSphere.00236-19 (PMC6483049; doi:10.1128/mSphere.00236-19)

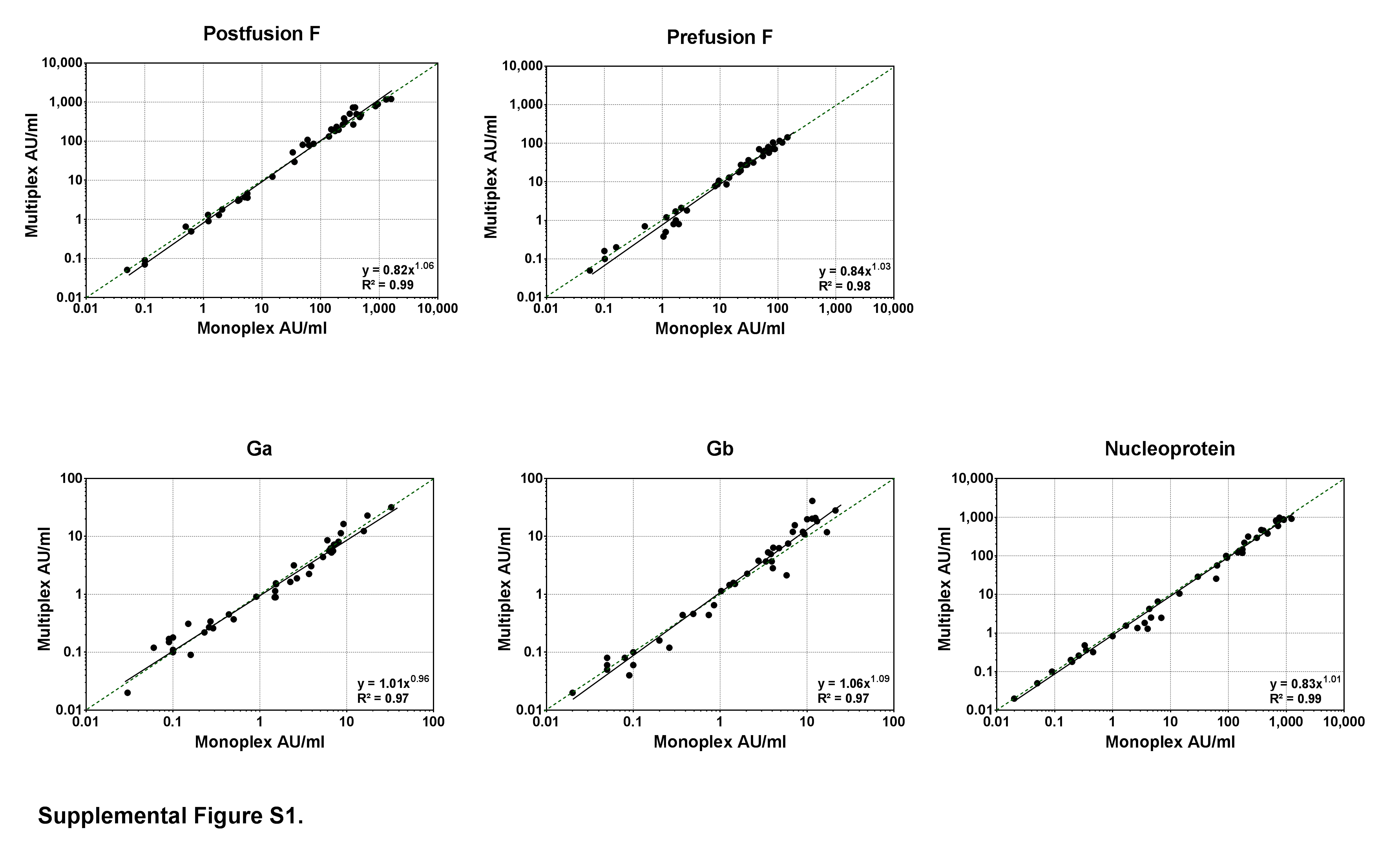

Supplement: FIG S1 [file mSphere.00236-19-sf001.tif]

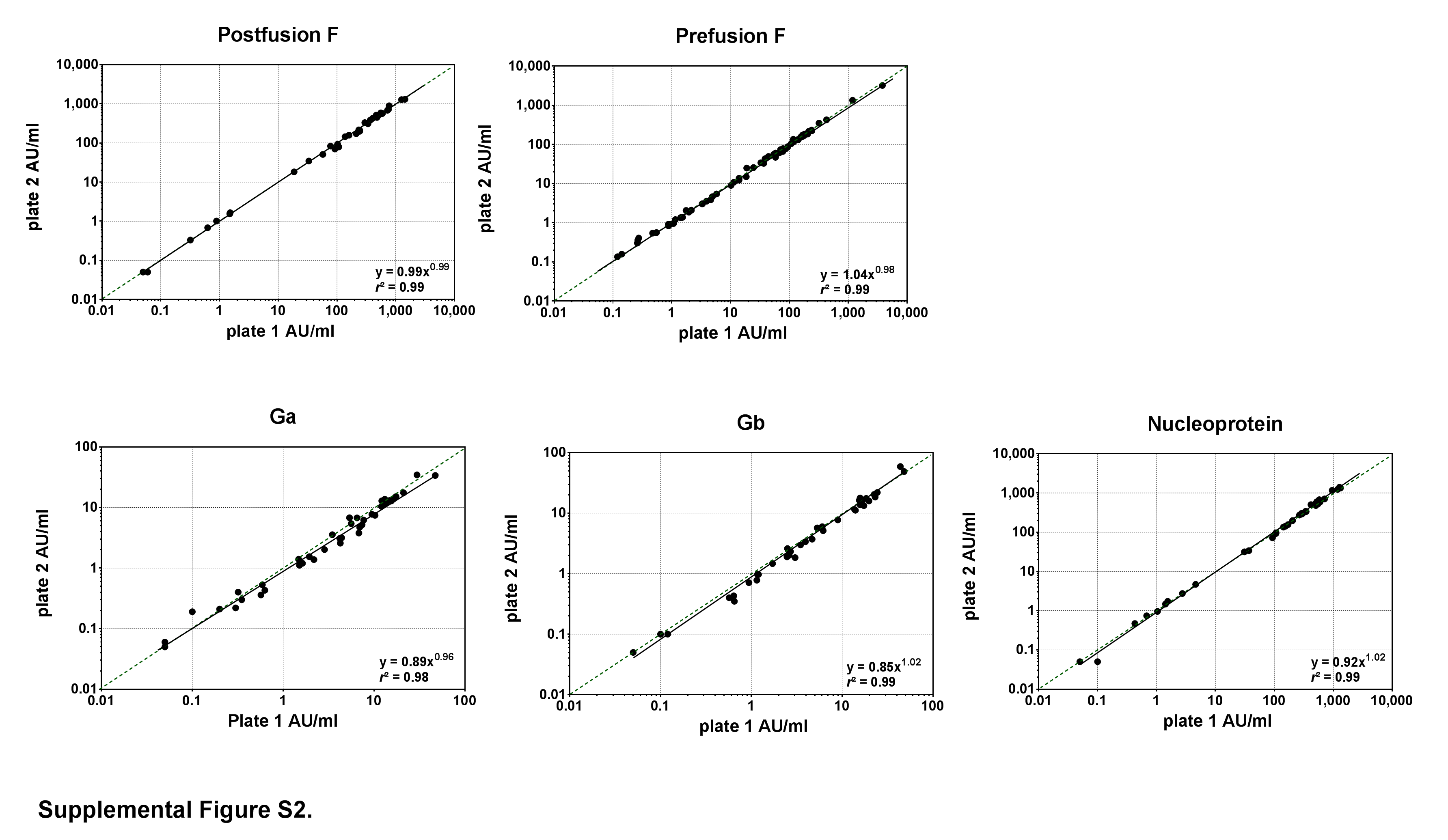

Supplement: FIG S2 [file mSphere.00236-19-sf002.tif]
