# Supplementary material for: Development and Standardization of a High-Throughput Multiplex Immunoassay for the Simultaneous Quantification of Specific Antibodies to Five Respiratory Syncytial Virus Proteins
Source: mSphere. 2019 Apr 24;4(2):e00236-19. doi: 10.1128/mSphere.00236-19 (PMC6483049; doi:10.1128/mSphere.00236-19)
Supplement: TABLE S1 [file mSphere.00236-19-st001.docx]

**Supplemental Table S1:** Characteristics of the monoclonal antibodies.

| **Monoclonal antibody** | **Species** | **IgG subclass** | **Target RSV protein** | **Antigenic site on F** |
| --- | --- | --- | --- | --- |
| **IVIG** | Human |  | All | - |
| **8262 clone 133-1H** | Murine |  | post/prefusion F | common epitope pre/postfusion F |
| **Biorad Mab** | Murine |  | post/prefusion F | common epitope pre/postfusion F |
| **8593 clone 63-10F** | Murine |  | post/prefusion F | common epitope pre/postfusion F |
| **8599 clone 131-2A** | Murine | IgG2a | postfusion F (± pre) | Site I |
| **56F** | Murine | IgG1 | post/prefusion F | common epitope pre/postfusion F |
| **Palivizumab** | Human | IgG1 | post/prefusion F | Site II |
| **D25** | Human | IgG1 | prefusion F | Site Ø |
| **AM14** | Human |  | prefusion F | Site V |
| **AM22** | Human |  | prefusion F | Site Ø |
| **5C4** | Murine |  | prefusion F | Site Ø |
| **MEDI 8897** | Human | IgG1 | prefusion F | Site Ø |
| **131-2G** | Murine | IgG1 | glycoprotein Ga/Gb | - |
| **021/19G** | Murine | IgG1 | glycoprotein Ga | - |
| **858-3 clone 130-12H** | Murine | IgG2κ | core protein N | - |
